# A new wild, pollinating bee species of the genus *Tetraloniella* from the Arabian Peninsula (Hymenoptera, Apidae)

**DOI:** 10.3897/zookeys.172.2648

**Published:** 2012-03-01

**Authors:** Abdulaziz S. Alqarni, Mohammed A. Hannan, Michael S. Engel

**Affiliations:** 1 Department of Plant Protection, College of Food and Agriculture Sciences, King Saud University, Riyadh 11451, PO Box 2460, KSA; 2 Division of Entomology, Natural History Museum, and Department of Ecology & Evolutionary Biology, 1501 Crestline Drive – Suite 140, University of Kansas, Lawrence, Kansas 66049-2811, USA

**Keywords:** Apoidea, Anthophila, Apinae, Eucerini, *Tetraloniella*, taxonomy, bees, Saudi Arabia, Qatar

## Abstract

A new species of the eucerine bee genus *Tetraloniella* Ashmead (Apinae: Eucerini) is described and figured from central Saudi Arabia and Qatar. *Tetraloniella (Tetraloniella) persiciformis* **sp. n.** is distinguished on the basis of coloration, integumental sculpturing, male metafemoral structure, and male terminalia. A floral record of *Pulicaria undulata* (L.) C.A. Mey. (Compositae) is noted for some of the material. Females superficially resemble those of *Tarsalia persica* (Warncke) (Ancylaini) in overall coloration but can be distinguished by the typical generic and tribal characters.

## Introduction

The bees of the widespread tribe Eucerini are notorious for their large numbers of similar species, with the distinctions even between genera being subtle and challenging to the melittologist. This is certainly true for the genus *Tetraloniella* Ashmead, in which distinctions with some *Synhalonia* Patton represent a significant obstacle to the taxonomist, not to mention ecologists, pollination biologists, and conservationists. Furthermore, even the validity of taxa such as *Melissina* Cockerell and *Xenoglossodes* Ashmead, today lumped as synonyms of *Tetraloniella* s. str. in a retrograde classification (LaBerge 2001; Michener 2007), highlights the challenges. It is, therefore, refreshing when remarkably distinctive members of this group are discovered and enhance our knowledge of variation within the larger lineage and expand our understanding of plant associations and biogeography. Along this line, we herein provide the description of a new species of *Tetraloniella* s. str. recently recognized from central and eastern areas of the Arabian Peninsula (Riyadh, Saudi Arabia and Qatar), and representing the first formal records of the subgenus from Saudi Arabia and Qatar.

## Material and methods

Material examined herein is deposited in the Plant Protection Department Museum of Insects, College of Food and Agriculture Sciences, King Saud University, Riyadh, Kingdom of Saudi Arabia (**PPDM**) and Division of Entomology (Snow Entomological Collections), University of Kansas Natural History Museum, Lawrence, Kansas, USA (**SEMC**). Morphological terminology follows that of Engel (2001) and Michener (2007). Photomicrographs were prepared with a Canon 7D digital camera attached to an Infinity K-2 long-distance microscope lens. Measurements were made with an ocular micrometer attached to an Olympus SZX-12 stereomicroscope.

## Systematics

### Genus Tetraloniella Ashmead. Subgenus Tetraloniella Ashmead

#### Tetraloniella (Tetraloniella) persiciformis sp. n.

urn:lsid:zoobank.org:act:B18D9320-0FA2-4730-A57C-C6C9D04E73D9

http://species-id.net/wiki/Tetraloniella_persiciformis

Figs 1 [Fig F2] –12

##### Holotype.

 ♂, 30.V.81 [30 May 1981], Riyadh, Saudi Arabia (SEMC).

##### Paratypes.

 Three total paratypes: 1♀, same data as holotype (SEMC); 1♀, Saudi Arabia, Riyadh, Al Amariah, [Mazra’ah] Majra Al-Gasim [farm], 6.vi.2011 [6 June 2011], M.A. Hannan // at flowers of *Pulicaria undulata* (PPDM); 1♂, Qatar, Al Sinnah, 14.ix.1979 [14 September 1979], C.G. Roche (SEMC).

##### Diagnosis.

**Figures 1–4.**
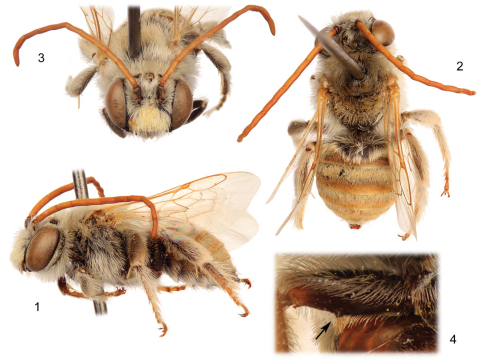
Male of *Tetraloniella persiciformis* sp. n. **1** Lateral habitus **2** Dorsal habitus **3** Facial view **4** Metafemur, with medioventral tubercle (arrow).

**Figures 5–9. F2:**
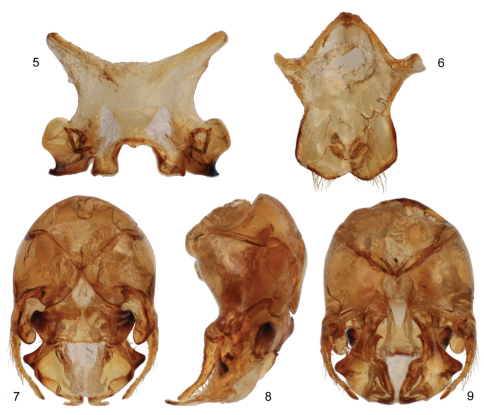
Male terminalia of *Tetraloniella persiciformis* sp. n. **5** Metasomal sternum VII **6** Sternum VIII **7** Genital capsule, dorsal view **8** Genital capsule, lateral view **9** Genital capsule, ventral view.

**Figures 10–12.**
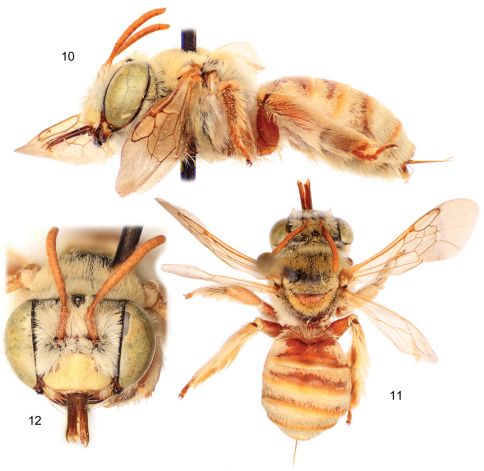
Female of *Tetraloniella persiciformis* sp. n. **10** Lateral habitus **11** Dorsal habitus **12** Facial view.

 The new species is structurally quite similar to the widespread *Tetraloniella (Tetraloniella) julliani* (Pérez) but differs by the lighter coloration (*Tetraloniella julliani* is largely black to dark brown throughout) and in the structure of the terminalia. The combination of the integumental and pubescence coloration (Figs 1–3, 10–12), presence of the metafemoral medioventral tubercle (Fig. 4), and male terminalia (Figs 5–9) also serve to distinguish the species from its congeners.

##### Description.

 ♂: Total body length 7.74 mm; forewing length 5.50 mm. Head wider than long, length 1.90 mm, width 2.73 mm; clypeus weakly protuberant, weakly and gently convex in profile; five maxillary palpomeres, ratio among them 1.0:0.87:0.40:0.27:0.27; antenna elongate, extending posteriorly to at least apex of disc of fourth metasomal tergum; first flagellomere 0.13 mm long, only slightly longer than pedicel, second flagellomere 6.6 times length of first flagellomere; length of second flagellomere 0.86 mm; length of third flagellomere 0.70 mm; apical five flagellomeres weakly crenulate; compound eyes converging below, slightly diverging above. Intertegular distance 1.83 mm. Metafemur with medial tubercle bearing small set of erect setae on ventral surface. Forewing with basal vein distad cu-a by about twice vein width; first submarginal cell shorter than combined lengths of second and third submarginal cells; first submarginal cell only slightly longer than third submarginal cell; anterior border of second submarginal cell slightly longer than anterior border of third submarginal cell; 1m-cu basad 1rs-m by about twice vein width; 2m-cu basad 2rs-m by about vein width; hind wing with 10–11 distal hamuli. Metasomal sternum VI medial extension with somewhat truncate apical margin, with narrow median longitudinal furrow on disc broadening slightly at its extreme apex, with paired strong carinae loosely paralleling lateral margins of median expansion, separated from actual sternal margin by less than one-quarter median ocellar diameter, carinae curving inward slightly and terminating at broadened opening of median furrow; terminalia as in figures 5–9 [note that sternum VII is similar to that of *Tetraloniella alticincta* (Lepeletier de Saint Fargeau), while the gonostylus is simple and decurved].

Integument of head largely shiny. Labrum and clypeus smooth with shallow coarse and contiguous punctures; face, vertex, and gena with small, contiguous irregular punctures, giving surface a roughened appearance except extreme borders with compound eyes smooth; postgena coarsely imbricate. Pronotum finely imbricate with sparse minute punctures; mesoscutum, mesoscutellum, and metanotum with shallow, contiguous, coarse punctures; pleura as on mesoscutum although punctures stronger; basal area of propodeum longitudinally rugulose giving way to coarse, irregular contiguous punctures; metasomal terga finely imbricate with small, well-defined punctures separated by less than a puncture width, in many areas contiguous or nearly so, extreme apical margins of terga impunctate and finely imbricate; sterna as on terga although punctures more coarse and shallow, and more widely spaced, particularly on more basal sterna.

Mandible yellow except dark brown at apex; labiomaxillary complex dark brown; labrum yellow; clypeus yellow; remainder of head black except scape and pedicel brown, flagellum yellowish brown; mesosoma black except tegula semitranslucent and light brown; legs dark brown except tarsi lighter brown, spurs pale yellow; wing membranes hyaline, veins light brown except those along costal margin darker; metasomal terga tending toward dark reddish brown in basal half, blending by midlength to brown and then to light, semitranslucent brown by apical margin, except tergum VII brown throughout; sterna reddish brown.

Integument largely obscured by pubescence except apically on metasomal terga and on sterna. Pubescence white to pale ochraceous, setae long, dense (typically obscuring much of integument), and plumose; metafemoral tubercle with patch of erect ochraceous setae, which extend in a line basad and become progressively shorter and less dense (Fig. 4); metasomal terga II–VI with erect to suberect setae, sparse setae, otherwise largely covered in shorter, appressed, plumose tomentum except along apical margins of terga III–VI and in apical half of tergum II where tomentum is replaced by short, appressed, simple setae; tergum I with appressed tomentum as on succeeding terga restricted to sub-apicolateral patches.

Female: As described for the male except in typical gender differences for eucerines and with the following modifications: Total body length 8.33 mm; forewing length 5.73 mm. Head wider than long, length 1.93 mm, width 3.13 mm; upper interorbital distance 1.88 mm, lower interorbital distance 1.61 mm. Intertegular distance 2.03 mm. Metafemur without ventral median tubercle; basimetatibial plate quite short, broadly rounded, largely obscured by setae from apex of femur. Sculpturing as in male except not as obscured by dense pubescence centrally on mesoscutum and anteriorly on mesoscutellum. Mandible yellow except apical half dark reddish brown with black apex; scape, pedicel, and flagellum yellowish brown; mesosoma dark reddish brown, ranging to black on mesoscutum, metanotum, and areas of pleura (areas of reddish brown greater one paratype than the other); legs reddish brown; metasoma reddish brown except apical margins of terga light brown and semi-translucent. Pubescence generally as in female except more ochraceous on vertex and dorsum of mesosoma than in male; scopal setae white on anterior surfaces, yellowish-orange on posterior surfaces and densely plumose.

##### Etymology.

 The specific epithet is a reference to the superficial resemblance of the female to *Tarsalia persica* (Warncke) (Ancylaini).

##### Comments.

 The Saudi female collected at Amariah in 2011 was captured between 8am–12pm and while sweeping on *Pulicaria undulata* (L.) C.A. Mey. (Compositae), known locally as [Jethjath]. The collection site was at the base of an elevated area near to Wadi Amariah and the highway to Riyadh.

## Discussion

It is interesting to note that the overall color pattern of this species is superficially similar to that of the wholly unrelated *Tetraloniella persica* (Warncke), known from Iran (Warncke 1979). Indeed, during an initial, quick sort of bee material collected from Amariah one of us (MSE) quickly placed the unassociated female as a possible *Tetraloniella persica* until the specimen was examined under a microscope and the true identity was revealed. Males are easily recognized as *Tetraloniella* while the females differ from the Ancylaini [refer to Engel et al. (2008) and ICZN (2010) for spelling] by the usual tribal differences [e.g., cu-a in hind wing moderately oblique at about one-half length of the second abscissa of M+Cu (second abscissa of M+Cu much shorter in Ancylaini); jugal lobe of hind wing about one-half vannal lobe (less than one-half vannal lobe in Ancylaini); long paraglossae (short in Ancylaini)].

With around 110 described species, *Tetraloniella* s.str. is one of the most diverse lineages in the Eucerini, second only to *Eucera* Scopoli. Merely 35 species are known from the New World and these have been recently revised, as have the approximately 32 sub-Saharan taxa (Eardley 1989) along with the four living in Madagascar (Pauly et al. 2001). The large and diverse Palaearctic fauna remains the greatest challenge and one of the more intractable problems given the ease in confusing species with *Synhalonia*.

## Supplementary Material

XML Treatment for Tetraloniella (Tetraloniella) persiciformis
